# Relationship between lumbar lordosis, pelvic parameters, PI-LL mismatch and outcome after short fusion surgery for lumbar degenerative disease. Literature review, rational and presentation of public study protocol: RELApSE study (registry for evaluation of lumbar artrodesis sagittal alignEment)^☆^

**DOI:** 10.1016/j.wnsx.2023.100162

**Published:** 2023-01-27

**Authors:** Fulvio Tartara, Diego Garbossa, Daniele Armocida, Giuseppe Di Perna, Marco Ajello, Nicola Marengo, Marco Bozzaro, Salvatore Petrone, Pietro Domenico Giorgi, Giuseppe Rosario Schirò, Simona Legrenzi, Davide Boeris, Andrea Piazzolla, Anna Claudia Passarelli, Alessandro Longo, Alessandro Ducati, Federica Penner, Flavio Tancioni, Alberto Bona, Giovanni Paternò, Cristina Tassorelli, Roberto De Icco, Giovanni Andrea Lamaida, Enrico Gallazzi, Giulia Pilloni, Elena Virginia Colombo, Paolo Gaetani, Enrico Aimar, Cesare Zoia, Roberto Stefini, Angelo Rusconi, Amos M. Querenghi, Carlo Brembilla, Claudio Bernucci, Andrea Fanti, Alessandro Frati, Antonio Manelli, Vitaliano Muzii, Mattia Sedia, Alberto Romano, Ali Baram, Silvia Figini, Elena Ballante, Giuseppe Gioia, Marco Locatelli, Mauro Pluderi, Carlotta Morselli, Roberto Bassani, Francesco Costa, Fabio Cofano

**Affiliations:** a Headache Science and Neurorehabilitation Center, IRCCS Mondino Foundation, Pavia, Italy; Department of Brain and Behavioral Sciences, University of Pavia, Italy; b Neurosurgery, Department of Neuroscience, A.O.U. Città Della Salute e Della Scienza, University of Turin, Italy; c Sapienza University of Rome, Policlinico Umberto I of Rome, Rome, Italy; d Spine Surgery Unit, Humanitas Gradenigo Hospital, Turin, Italy; e Orthopedics and Traumatology Unit, ASST Grande Ospedale Metropolitano Niguarda, Milan, Italy; f Neurosurgery Unit, ASST Grande Ospedale Metropolitano Niguarda, Milan, Italy; g Department of Neuroscience and Organs of Sense, Orthopaedics Section, Faculty of Medicine and Surgery, University of Bari, Bari, Italy; h Spine Surgery Unit, Humanitas Cellini Hospital, Turin, Italy; i Neurosurgery, Istituto Clinico Città Studi, Milan, Italy; j Chirurgia Vertebrale, Piccole Figlie Hospital, Parma, Italy; k Scoliosis and Vertebral Orthopedics and Traumatology Unit, ASST Gaetano Pini - CTO, Milan, Italy; l Neurosurgery, ASST Fatebenefratelli-Sacco, Milan, Italy; m Vertebral Surgery Unit, Città di Pavia Clinic, Pavia, Italy; n Neurosurgery, IRCCS Policlinico San Matteo Foundation, Pavia, Italy; o Neurosurgery Unit, ASST West Milan, Legnano, Italy; p Spine Surgery Unit, Humanitas San Pio X Hospital, Milan, Italy; q Neurosurgery Unit, ASST Papa Giovanni XXIII, Bergamo, Italy; r Neurosurgery, Policlinico Santa Maria Alle Scotte, University of Siena, Italy; s Spine Neurosurgery, Salus Hospital, Reggio Emilia, Italy; t Neurosurgery, Humanitas Istituto Clinico Catanese, Catania, Italy; u Department of Neurosurgery, Humanitas Research Hospital, Rozzano, Milan, Italy; v Statistics, Department of Political and Social Sciences, University of Pavia, Italy; w Vertebral Surgery Unit, Piccole Figlie Hospital, Parma, Italy; x Neurosurgery, Fondazione IRCCS Ca’ Granda, Ospedale Maggiore Policlinico di Milano, Italy; y II Spine Unit Milan, Italy, IRCCS Galeazzi Orthopedic Institute, Milan, Italy; z Spine Surgery Unit - NCH4 - Department of Neurosurgery - Fondazione IRCCS Istituto Nazionale Neurologico “C. Besta”, Milan, Italy

**Keywords:** Spine, Neurosurgery, Fusion surgery, Lumbar degenerative disease, Lumbar lordosis, Pelvic Index (PI), Lumbar Lordosis (LL), transforaminal interbody fusion (TLIF), posterior interbody fusion (PLIF), antero-lateral interbody fusion (ALIF), latero-lateral interbody fusion (LLIF), segmental lumbar lordosis (LS), Oswestry disability index, Short Form-12 (ODI-12), body mass index (BMI)

## Abstract

**Background:**

Vertebral arthrodesis for degenerative pathology of the lumbar spine still remains burdened by clinical problems with significant negative results. The introduction of the sagittal balance assessment with the evaluation of the meaning of pelvic parameters and spinopelvic (PI-LL) mismatch offered new evaluation criteria for this widespread pathology, but there is a lack of consistent evidence on long-term outcome.

**Methods:**

The authors performed an extensive systematic review of literature, with the aim to identify all potentially relevant studies about the role and usefulness of the restoration or the assessment of Sagittal balance in lumbar degenerative disease. They present the study protocol RELApSE (NCT05448092 ID) and discuss the rationale through a comprehensive literature review.

**Results:**

From the 237 papers on this topic, a total of 176 articles were selected in this review. The analysis of these literature data shows sparse and variable evidence. There are no observations or guidelines about the value of lordosis restoration or PI-LL mismatch. Most of the works in the literature are retrospective, monocentric, based on small populations, and often address the topic evaluation partially.

**Conclusions:**

The RELApSE study is based on the possibility of comparing a heterogeneous population by pathology and different surgical technical options on some homogeneous clinical and anatomic-radiological measures aiming to understanding the value that global lumbar and segmental lordosis, distribution of lordosis, pelvic tilt, and PI-LL mismatch may have on clinical outcome in lumbar degenerative pathology and on the occurrence of adjacent segment disease.

## 1 Introduction

Degenerative pathology of the lumbar spine is widespread, affecting approximately 5.7% of the European population.^1^ Lumbar arthrodesis, with its different technical options, is a commonly adopted surgical therapy accounting for 30,000 procedures performed annually in Italy and over 450,000 in the United States.^2^ However, lumbar vertebral arthrodesis for degenerative disease still remains burdened by clinical problems with significant negative results, including lack of clinical improvement, late symptoms relapse, or clinical worsening.^3^ The evidence-based medicine on this topic is still insufficient and generally does not exceed evidence class B.^4^^,^^5^

The available guidelines are mainly based on level II studies^6^, ^7^, ^8^, ^9^, ^10^, ^11^, ^12^ and only offer recommendations that may support practitioners in clinical activity.^5^ Thus, surgical treatment of lumbar degenerative disease remains extremely heterogeneous, considering the number of technical options available for the single pathology. Lack of standard of care leads to treatment strategies based on institutional, departmental, or personal experience planned on inconsistent scientific evidence.^13^

The introduction in the clinical practice of the sagittal balance assessment with the evaluation of the meaning of pelvic parameters and spinopelvic mismatch offered new evaluation criteria for lumbar degenerative pathology^14^^,^^15^ and the outcome of short lumbar arthrodesis surgery.^16^, ^17^, ^18^

Restoration of Sagittal alignment and Pelvic Index (PI)-Lumbar Lordosis (LL) mismatch is closely associated with a better outcome in spinal deformities.^19^ However, few studies in the literature report the impact of sagittal balance assisment on patients' post-surgical outcome.

At the same time, there is still a lack of consistent evidence regarding short-segment arthrodesis for lumbar degenerative pathology.

The definitive value for lumbar degenerative pathology of these aspects of the surgical outcome remains to be clarified without consolidated evidence. We conducted a comprehensive review of literature about this topic and here we propose our upcoming prospective study. The RELApSE study is, to our knowledge, the first prospective and multicenter study on these topics. We present the study protocol registered on trials.gov (NCT05448092 ID, protocol ID 012022) and discuss the rationale through a comprehensive literature review.

## 2 Methods

### 2.1 Background

Interbody fusion, including: transforaminal (TLIF), posterior (PLIF), anterior (ALIF), and lateral (LLIF); effectively treat lumbar degenerative pathology and provide spinopelvic balance and the impact of the interbody approach on segmental and adjacent level lordosis could be an important factor to consider during pre-operative planning to achieve pre-specified alignment goals.

**Fig. 1:**
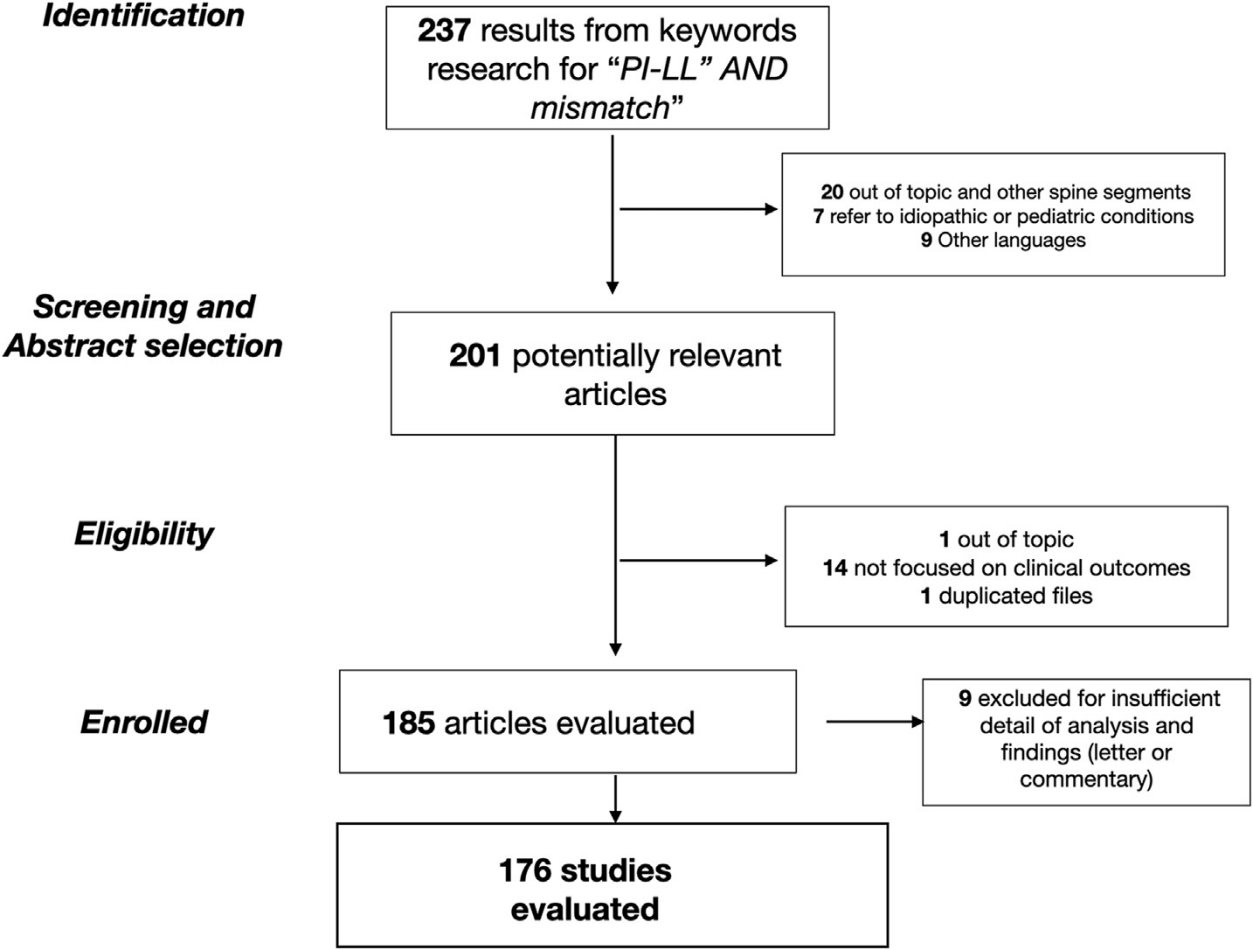
The flow-chart showing the selection according to PRISMA criteria of the articles used for the review.

From the literature search conducted (Fig. 1), 176 papers dealing with this topic were selected, and after careful analysis, we found that the results are often conflicting with each other and often incomplete. Over the past ten years, many studies reported associations between PI-LL mismatch, reduced lumbar lordosis, increased pelvic tilt, and outcome of lumbar arthrodesis for degenerative lumbar disease.^16^^,^^20^, ^21^, ^22^, ^23^, ^24^ Other authors, on the other hand, reported an absence of correlation between the same parameters and clinical outcomes.^25^^,^^26^ In addition, several authors have been reported evidence regarding association of adjacent level disc degeneration and elevated pelvic tilt, persistent PI-LL mismatch and altered LL4-S1/LL ratio.^18^^,^^27^, ^28^, ^29^, ^30^, ^31^, ^32^, ^33^, ^34^, ^35^, ^36^ Also, on this aspect, other studies identify different elements as predisposing factors for junctional pathology.^37^^,^^38^

## 3 Review of literature

The English literature is systematically investigate using MEDLINE, the NIH Library, Pubmed, web of science and Google Scholar according to the Preferred Reporting Items for Systematic Reviews and Meta-analysis (PRISMA) guidelines, with the aim to identify all potentially relevant studies about the role and usefulness of the restoration or the assessment of Sagittal balance in lumbar degenerative disease. Searching for relevant studies, the reference section of included articles was analyzed.

The search was performed typing the following items (Pelvic incidence) AND (mismatch) (Pelvic incidence-lumbar lordosis) AND (mismatch) (PI-LL) AND (mismatch) (PI-LL) AND (surgical assessment) (Pelvic incidence-lumbar lordosis) AND (surgical assessment), obtaining 237 potentially titles.

The first step of selection was focusing the usefulness of the study of Sagittal balance parameters in lumbar degenerative disease. In this regard, we chose to exclude by title and abstract papers that dealt with other segments of the spine, non-degenerative conditions such as congenital and pediatric forms, and discarding publications that had other languages outside of English, obtaining through these initial inclusion criteria 201 potentially relevant articles. As further criterion of inclusion, we chose to consider adult populations with just degenerative conditions (excluding post-traumatic disease). It is worthwhile note that neurosurgical intervention was not considered as a criterion of inclusion itself since both patients eligible for neurosurgical treatment and non-surgical patients has been included. Given these premises, we selected papers according to the following inclusion criteria.

- Availability of full-text articles
- English text only
- Patients older than 18-year-old without history of trauma
- Use of Sagittal balance parameters to select, plan or evaluate the treatment;
- Presence of neurological outcome evaluation. No specific limitation was applied regarding the timing of neurological evaluation after treatment.
- Papers published from 1985 onwards (for availability of recent imaging studies such MRI) Conversely, exclusion criteria were:
- Full-text articles in languages other than English
- Studies reporting patients with post-traumatic disease, other spinal segment considered
- Patients younger than18-year-old
- No data available about neurological outcome or not focused on clinical outcome

Data extracted from each study were (1) authors, (2) year of publication, (3) study design, (4) purpose of the study, (5) disease condition, (6) number of patients included, (7) PI-LL parameters used, (8) lumbar spine segment evaluated, (9), neurological functions evaluated (10) clinical outcome (11).

### 3.1 Results

A total of 237 studies were found through PubMed database search and reference section screening. Duplicates check was carried out by an automatic tool working on Microsoft Excel spreadsheets. Out of the initial papers, 1 duplicate, 20 were out of topic identifiable by the title, 9 with other languages, were removed; thus 201 titles with abstract were identified and, following the eligibility criteria, 185 papers were screened. A further qualitative skimming led to articles being selected for full text screening, out of which a total of 176 articles were included in the systematic review (Fig. 1).

### 3.2 Study design

The study aims to evaluate the relationships between radiological data and patients' reported outcome. Starting from a heterogeneous population in terms of clinical conditions, pathology, and surgical treatment options, the study method is to make the population homogeneous on some data available in all patient's end that can be analyzed independently. These data are pelvic parameters (pelvic incidence, pelvic tilt, sacral slope), segmental lumbar lordosis (LS), global lumbar lordosis (LL), PI-LL mismatch, and L4-S1/LL lordosis ratio; clinical results based on administered questionnaires (Oswestry disability index, Short Form-12, ODI-12) and overall outcome assessment at FU (6 point scale: excellent (entirely resolved symptoms), good (good clinical improvement, minor signs), fair (improvement compared to preoperative but still with relevant symptoms), unchanged (symptoms similar to preoperative), negative (worsening of symptoms compared to preoperative); severely worsened (reduction of personal autonomy compared to preoperative due to neurological deficits); occurrence of symptomatic junctional pathology (yes/no), need for surgical revision of the operated level (yes/no) or of the adjacent level (yes/no).

### 3.3 Endpoints

Primary end-point: analysis of the relationship between clinical results (ODI, SF-12, global outcome) and pelvic parameters, overall lumbar/segmental postoperative lordosis/ratio L4-S1-overall lumbar lordosis (delta pre-postoperative-follow-up values) in the hypothesis that the persistence of elevated PI-LL mismatch, reduced LL or altered pelvic parameters (pelvic tilt) may represent independent adverse prognostic factors for patients reported outcome, the occurrence of symptomatic junctional pathology and need for surgical revision.

Secondary end-points: 1) Comparison between different interbody fusion techniques (Anterior Lumbar Interbody Fusion (ALIF) vs. Posterior Lumbar Interbody Fusion (PLIF) vs. Transforaminal Lumbar Interbody Fusion (TLIF) vs. Extreme lateral Lumbar Interbody Fusion (XLIF)) in terms of increasing segmental lordosis; 2) Comparison between different interbody fusion techniques (ALIF vs. PLIF vs TLIF vs. XLIF) in terms of changing the global lumbar lordosis, spinopelvic parameters and PI-LL mismatch; 3) Long-term evaluation of lumbar lordosis stability in relation with occurrence of subsidence or pedicular screws failure.

### 3.4 Sample size and statistical analysis

The sample size is assessed in relation to the primary objective. We want to compare the variation in clinical parameters before and after the intervention (ODI, SF-12: MCS, PCS, global outcome) between the groups finally identified by the presence/absence of LL-PI mismatch.

In the hypothesis of no difference, the data necessary for the calculation will be referred to as reported by Divi et al^26^ As regards MCS-12, the study says a delta equal to 4.1 in subjects without mismatch and equal to 4.1 in issues with mismatch, consequently, no difference is expected, and this parameter is not included in the calculation of sample size. Concerning ODI, the study going to reports a delta equal to −22.8 in subjects without mismatch and equivalent to −20.9 in subjects with a mismatch (hypothesized sd equal to 5). For PCS-12, the study will reports a delta equal to 9 in subjects without mismatch and similar to 10 in subjects with a mismatch (hypothesized sd equal to 2).

The significance is appointed at 0.05, a test power of 0.95, assuming to compare the two samples using a two-tailed *t*-test with a minimum sample size of 362 subjects is required. Since the study is multicentric and considering the risk of loss of information during follow-up or the presence of incorrect preoperative radiographic examinations, the enrollment of a total of 500 patients is considered exhaustive. The evaluation of the secondary end-points, which is not essential, will be carried out on the same population sample.

Quantitative variables will be expressed using means and standard deviations (medians and quartiles when appropriate). A comparison of proportions will be performed with the Chi-squared test for categorical variables. Qualitative data will be expressed as a raw number and a population percentage. Continuous variables will be compared using the Student *t*-test and ANOVA (or non-parametric analog). The correlation between numerical variables will be evaluated through Pearson's correlation coefficient and relative test (or similar Spearman coefficient and test, if appropriate). Any linear or non-linear regressions will be assessed for the relationship between lumbar lordosis delta, pelvic parameters, and clinical improvement. Depending on the results provided by the analyzes described, it may be appropriate to set up mixed-effects models to assess the longitudinal trend of the study parameters. The possible implementation of non-parametric models of supervised machine learning (e.g., random forest, SVM) will be evaluated to verify the actual predictive power of the variables under consideration concerning the clinical evaluation.

Due to the nature of the multicentre study and the high sample size, it is planned to create an interactive report (dashboard) for viewing the statistical results obtained.

### 3.5 Surgeons’ identification criteria and patients recruitment

The participant's investigators will be identify in the Italian context among orthopedics or neurosurgeons with proven experience in the field of vertebral surgery (based the selection concerning the number of year, number of procedures done by the surgeon) by direct invitation from one of the steering committee members. Surgeons interested in the study must sign a letter of intent to underline the commitment required and the roles of the investigator. Each participating investigator is required to obtain approval from their relevant ethics committee. Investigators will recruit patients in the context of their regular clinical and surgical activity. Recruitment must be prospective, and the patient must be enroll before surgery to avoid selection bias related to clinical results. Each participating surgeon will be required to recruit a minimum of 20 complete and evaluable patients to be included in the investigators, while the number of patients enrolled should not exceed 60 cases for the single surgeon with the objective to homogenize the contribution of individual investigators and avoiding, within the enrolled population, excessive imbalances and discrepancies. Once the programmed sample size has been reached, recruitment will be close. Eventually for some centers and some surgeons, consideration will be given to stratifying patients as private, insured or government-funded and whether they have claims or demands.

### 3.6 Patients inclusion, exclusion, and withdrawal criteria

All patients with the following requirements will be included in the observational study.

1. Patients undergoing to an instrumented lumbar arthrodesis operation at 1, 2, or 3 levels
2. Age between 18 and 75 years
3. Agree to inclusion in the study with a subscription of informed consent, available for five years follow-up, including phone interviews.
4. Availability of adequate preoperative radiological documentation: CT or MRI of the lumbar spine; standing lumbar spine x-ray performed in a neutral position in which the pelvic parameters (pelvic incidence, pelvic tilt) and all lumbar segments can be correctly assessed
5. Availability of adequate and comprehensive clinical information, including the presence of preoperative ODI and SF-12
6. Availability of adequate information regarding surgery
7. Availability of postoperative radiological documentation: lumbar spine X-ray with the exact requirements as point 4 performed in the postoperative period and at one year follow-up.
8. Availability of adequate and comprehensive clinical information, including ODI score and SF-12 questionnaire at follow-up. The minimum follow-up for each patient included must be 12 months, to be continued for a total of 5 years.

Patients who, although already enrolled, do not meet the inclusion criteria at final evaluation will be excluded. Patients with a life expectancy of fewer than 5 years due to associated diseases must be excluded from the study.

Patients who, for various reasons, have to withdraw their consent to be included in the study will also be excluded. There are no other exclusion criteria.

### 3.7 Data collection

For the recruited patients, all the following data are collected.

- general: age, sex, smoking habit, previous diagnosis of osteoporosis
- surgical data: type of surgery performed, kind of arthrodesis performed, date of surgery, used cages material, used cage lordosis degrees, intraoperative adverse events, ODI, technical errors, complications, hardware failure, need for reoperation, the occurrence of junctional pathology, intervention-junctional pathology time
- clinical data: diagnosis (type of degenerative disease), duration of symptoms, presence of claudication or radicular pain, presence of neurological deficits, presence of L5 sacralization, ODI score, SF-12 PCS, SF-12 MCS, days of postoperative hospitalization, overall outcome assessment. ODI score and the SF-12 questionnaire (general health status) are used for outcome evaluation.
- radiological data: overall lumbar lordosis, segmental lordosis of all lumbar levels, pelvic incidence, pelvic tilt, sacral slope. The values will be calculated according to the criteria reported by Duval-Beaupère. From these data, theoretical pelvic tilt will be subsequently calculated regarding Vialle's formula (PT = PI x 0.37–7), pelvic delta tilt (PT - theoretical PT), PI-LL mismatch (expressed on the calculated ideal lordosis such as PI ​+ ​10°), L4-S1 lordosis/LL lordosis (percentage). The vertical sagittal axis (SVA C7–S1) is not considered in this study about the need to obtain teleradiography of the entire spine, a non-routine and mainly not required examination for degenerative diseases.

All images must be centralized for measurements anonymously and coded. All radiological data, measured as angles or derived numbers, will be calculated by independent data managers recruited as volunteers among medical specialists or fellows (see Study Board). The angle values will be calculated by performing three successive measurements on the same radiological image by three independent examiners not involved in the surgical management. Subsequent calculation of the average of the nine values is obtained. Calculating pelvic angles and parameters can be done with freeware SurgimapÒ software developed by a group of vertebral surgeons and engineers to support anatomical evaluation and surgical planning (Nemaris Inc.TM innovation, New York, NY, USA). The software can be downloaded free from the website www.surgimap.com where all the policies and conditions of use are visible.

### 3.8 Follow-up

All patient follow up continues, unless the patient withdraws consent explicitly, for the entire study duration initially planned for five years.

The follow-up includes postoperative clinical evaluation three months after surgery, one year and up to 5 years annually. Any additional assessment will be dictated only by clinical needs and must be added to the follow-up with a related time of occurrence. The follow-up evaluations must include acquiring all clinical data, including ODI and SF-12 evaluation questionnaires. Follow-up is IN the responsibility of a single investigator. A telephone assessment may be performed on patients who are not available for outpatient assessments or who cannot be reached by completing ODI and SF-12 by interview.

The follow-up must also include the acquisition of a lumbar spine standing X-ray at three months and 12 months, as normally suggested by good clinical practice. According to the literature, implant settlement and cages subsidence occur mainly in the first 12 months.^39^^,^^40^^,^^90^ Further or subsequent radiological examinations will eventually be performed exclusively for clinical needs (persistent symptoms, clinical worsening) and must be added to the study data.

### 3.9 Ethical concern

RELApSE is a purely observational study registered and published on trials.gov with NCT05448092 ID (protocol ID 012022). No interference is foreseen on the patient's diagnostic-therapeutic path or technical treatment options chosen by participating surgeons. Furthermore, no form of experimentation with techniques or materials is envisaged. Data collection is prospective in the context of regular clinical activity. The study is not sponsored. The management of privacy and personal data is in full compliance with the current Italian law. The study guarantees the transmission and collection of data in a completely anonymous form. The study is observational and not sponsored and only represents an analysis and comparison of data obteined from regular clinical and surgical activity. No experimentation is envisage. There are no conflicts of interest for any of the board members.

### 3.10 Study monitoring and supervision

The supervision of the study provides for the verification of the patient's record completeness, the adequacy of the radiographs for the purpose of the study, and the correct presence of follow-ups. Random evaluation of the accuracy of the measurements. Evaluation of proper data analysis procedures. Participation and supervision of the results analysis. Supervision is carried out by independent figures who are not part of the study board and are not investigators of the study.

A telephone evaluation will be performed anonymously on a sample of enrolled patients representative of all investigators to verify the data's accuracy and truthfulness.

## 4 Discussion

The latest AANS guidelines on arthrodesis for degenerative pathology (part 7–12, 2014)^6^, ^7^, ^8^, ^9^, ^10^, ^11^ report grade B and C recommendations based on evidence level II, III, and IV studies. Furthermore, all these chapters reported, “There is no evidence that conflicts with the previous recommendations published in the original version of the Guidelines” (Guidelines for the performance of fusion procedures for degenerative disease of the lumbar spine, 2005). There are no observations in the guidelines about the value of lordosis restoration or PI-LL mismatch. Therefore, between 2005 and 2014, there has not been a significant evolution of scientific evidence in this field of vertebral surgery. Therefore, the evaluation of the sagittal balance and related parameters could likely represent a new area of discussion and a possible way to generate some further criteria for evaluating the outcome of surgical treatment for lumbar degenerative disease. This could also help to create some evidence for lumbar arthrodesis. Afterward, an increasing number of publications have reported on the role of lumbar lordosis, pelvic parameters, and PI-LL mismatch.

A recent systematic meta-analysis highlighted a strong relationship between LBP and reduced lumbar lordotic curve mainly when patients were analyzed with age-matched healthy controls.^15^ In 2000 Lazennec et al firstly analyzed the relationship between radiological parameters and postfusion pain, focusing attention on the vertical position of the sacrum.^17^ Patients with pain persisting after arthrodesis showed both reduced SS and increased PT, with PT reaching almost twice the normal value. Authors postulated that “achieving a strong fusion should not be the only goal. Appropriate position of the fused vertebrae is also paramount to minimize muscle work during posture maintenance”.^20^, ^21^, ^22^ An increased LL and SL was associated with better outcome (VAS, ODI) after unilateral instrumented TLIF for single-level lumbar degenerative disease^41^, ^42^, ^43^ and after 1 level^43^ or 2-level PLIF for 2-level (L3-L4 and L4-L5) degenerative spondylolisthesis.^20^ Hioki et al described a positive linear correlation between the increase in lordotic angle and improvement of JOA score at outcome evaluation after two-level PLIF.^22^

Furthermore, postoperative reduction of pelvic tilt was associated with better outcomes after PLIF surgery for spondylolisthesis^24^^,^^44^^,^^45^ and sacral slope increased to more than 30° after single-level TLIF.^43^ The review by Le Huec et al suggested that the increase in PT after surgery is associated with significant low back pain. At the same time, the restoration of a regular PT results in an excellent clinical outcome.^18^ The study of Aoki et al was the first investigating influence of PI-LL mismatch on postoperative residual symptoms after 1 or 2 levels TLIF.^16^ More considerable PI-LL mismatch was significantly associated at one year follow-up with VAS for LBP, leg pain, and leg numbness but not with postoperative disability (ODI). Detailed VAS analysis highlights the association of mismatch with standing low back pain but not with LBP while sitting or in motion. Worse ODI scores were associated at 2 years follow-ups with PI-LL mismatch after four-level lumbar (L2-S1) fusion surgery^46^; moreover, significant correlations between PI-LL mismatch and improvement in both JOA score-VAS for LBP at two year follow up has been described for patients with degenerative lumbar scoliosis treated with short-segment fusion (1, 2 or 3 level TLIF) at the affected levels.^47^ Radovanovic et al reported a better patient-reported outcome, after surgery for degenerative spondilosthesys, in patients with SVA less than 50 mm. Patients with an SVA ≥50 mm presented reduced lumbar lordosis with increased mismatch and had a worse SF-36 PCS and Oswestry Disability Index (ODI; *p* = 0.043) as well as more back pain.^21^

Better improvement of PI-LL mismatch with reduced PT and higher LL was found in patients without residual back pain after the OLIF procedure,^48^ and similarly a significant linear association with ODI was reported for independent variables LL, delta LL, and PI-LL status after Interbody fusion for degenerative disc disease.^49^ A cutoff value of 27,5° for preoperative PI-LL mismatch is reported as a negative factor for outcome of patients who underwent second PLIF surgery for ASD and as a predisposing factor for subsequent long corrective surgery.^23^ Finally, sagittal malalignment with PI-LL mismatch greater than 10° was also associated with the occurrence of pseudarthrosis.^50^

On the contrary, Hsu et al reported no correlation between LL or LL restoration ratio and the outcome of patients undergoing PLIF for degenerative spondylolisthesis,^51^ while Jia et al concluded that PI-LL mismatch is not associated with clinical outcome (VAS, ODI) after MIS-TLIF for lumbar stenosis.^25^ A comparative study between ALIF and PLIF also showed no relationship between LL and patients outcome.^52^ A 2017 systematic review by Rhee et al selected only 4 articles for final statistical analysis and pointed out the lack of well-powered studies on this topic. No statistically significant improvement in both ODI and VAS was related to the restoration of segmental lordosis. So the correction of malalignment does not seem to yield clinical improvements for short lumbar arthrodesis.^53^

A recent large retrospective study shows that patient outcomes in short-segment lumbar fusion for the degenerative lumbar disease are equivalent in patients with and without a postoperative PI-LL mismatch at one year follow-up. The two groups' preoperative, postoperative, or delta outcome scores (PCS-12, ODI, VAS back, VAS leg) were noted. PI-LL mismatch was not found to be an independent predictor for patient-reported outcome on multivariate analysis (*P* > 0.05). This study suggests that limited surgery addressed to focal neurological disease has equivalent effects of corrective surgery.^26^

Adjacent segment degeneration (ASD) after lumbar arthrodesis, both symptomatic and radiological, is a well-known problem. Many studies discuss this topic, and several aspects were analyzed as causes or risk factors. A prospective study by Ekman shows that surgical fusion accelerates the occurrence of degenerative discopathy at the adjacent level compared with natural history.^54^ Incidence has a wide range between 2,62 and 84% with a prevalence of proximal level and main associated factors were old age, body mass index (BMI), previous degenerative disc o facet disease, type of pathology, multiple-level fusion, male, intraoperative superior facet joint violation, laminectomy, sagittally oriented facet joint angle, PLIF and progressive fatty degeneration of the multifidus muscle.^54^, ^55^, ^56^, ^57^, ^58^, ^59^, ^60^, ^61^, ^62^, ^63^, ^64^, ^65^

In 2001 Kumar et al firstly reported a significant association between ASD and the C7 sagittal plumb line. A vertical sacrum was highly associated with ASD even with regular C7 plumb line. Sacral inclination was considered an essential aspect of sagittal alignment as an expression of the compensation mechanism.^66^ Several authors reported a correlation between ASD and reduced lumbar lordosis as a significant independent factor^27^^,^^67^, ^68^, ^69^ or associated with other factors.^57^^,^^70^, ^71^, ^72^ Also, reduced postoperative segmental lordosis was associated with the occurrence of ASD.^29^^,^^70^^,^^73^^,^^74^ Soh et al and Bae et al suggested that the most important factor for the prevention of ASD is the restoration of segmental lordosis.^29^^,^^73^ Kim KH et al described association between reduced segmental lordotic angle and symptomatic ASD in isolated L4–L5 spondylolisthesis treated with interbody fusion and pedicle screw fixations.^75^ Again the pre-postoperative segmental lordosis delta was reported as a significant risk factor for ASD, especially with early onset.^50^^,^^76^

The relationship between pelvic parameters and ASD was also studied. Nakashima et al identified a high degree of pelvic incidence as a risk factor for early-onset radiological ASD, probably in relation to the reduced probability of obtaining appropriate lordosis after surgery.^28^ This association was also confirmed by a recent meta-analysis.^69^

Some authors found a significant reduction of sacral slope angle in patients with ASD.^18^^,^^30^ Similarly, Di Martino et al reported in ASD patients significantly lower SS and consequent higher PT values related to pelvic retroversion and hyperlordosis compensation mechanism.^31^ In this study, the Authors defined SS value below 39° or PT above 21° as a vital risk factor for symptomatic ASD (relative risk 1.73 for SS and 3.663 for PT). Another study confirmed that a PT greater than 24.1° could be considered predictive of ASD after lumbar TLIF.^77^ Also, preoperative PT with a 22.5° cutoff was strongly associated with ASD (risk 5.1 greater).^32^ A meta-analysis by Phan et al concludes that the development of ASD may be predicted from the evaluation of Spinopelvic alignment parameters (PT, SS, PI-LL mismatch, and LL) in patients with lumbar fusion for degenerative disease.^78^ An elevated PI-LL mismatch has been reported to be closely associated with the development of symptomatic ASD^33^^,^^68^ or radiological ASD.^46^ A PI-LL mismatch greater than 15° was identified as a significant independent risk factor for radiographical ASD in patients with L5-S1 spondylolytic spondylolisthesis treated with single-level PLIF.^34^ Moreover Wang et al reported a strong association between symptomatic ASD after lumbar fusion and more significant PI-LL mismatch but identified different PI-LL mismatch cutoffs in patiens below 60 years (PI-LL >10°) and older patients (PI-LL > 20°) to reach statistical significance. The authors hypothesize that the ideal correction of LL may vary with increasing age.^79^ Rothenfluh et al reported 10-times higher risk of ASD occurrence for patients with elevated PI-LL mismatch (>10°).^35^ Patients with ASD have higher PI, higher PT, and lower lumbar lordosis.The authors conclude that when fusion surgery is performed without treatment of intrinsic deformity and PI-LL mismatch the occurrence of ASD can be expected. Finally lumbar distribution index (LDI = L4-S1 lordosis/lumbar lordosis x 100) was strongly associated with the occurrence of ASD: patients with reduced distal L4-S1 lordosis and consequent low LDI present more significant risk of developing ASD.^36^ Kim et al reported frequent occurrence of ASD in patients with LDI less than 50% also when PI–LL was satisfactorily corrected to less than 10°.^80^ Obtaining an appropriate postoperative LDI in L4-S1 may have a crucial role in preventing ASD. In both clinical analysis and mechanistic simulation environments the increased loading and biomechanical shear forces at fusion adjacent level have been postulated and discussed for patients with PI-LL mismatch. These experimental data offer a different element in evaluating the association between ASD occurrence and sagittal malalignment.^81^^,^^82^

On the contrary, some authors have highlighted the absence of a relationship between ASD and sagittal alignment. Anandjiwala et al reported that LL is not a risk factor for ASD occurrence,^83^ while Chen et al reported no differences in lumbar lordosis between patients presenting ASD and those who did not.^84^ Masevin et al analyzing the risk factors for ASD, reported the absence of a role of the sagittal balance in short fixations for which the only risk factor is preoperative degenerative changes.^85^ A meta-analysis by Wang et al, based on 19 papers, showed that postoperative PT and SS are not associated with ASD occurrence.^69^ Multivariate analyses showed that segment distraction was the most significant risk factor after L4-L5 PLIF^86^ or only multilevel surgery associated with high rate reoperation.^87^ Finally, a recent study on the prognostic factors of ASD after L4-L5 fusion does not consider sagittal alignment at all, suggesting that many surgeons still underestimate these aspects.^88^

**Fig. 2:**
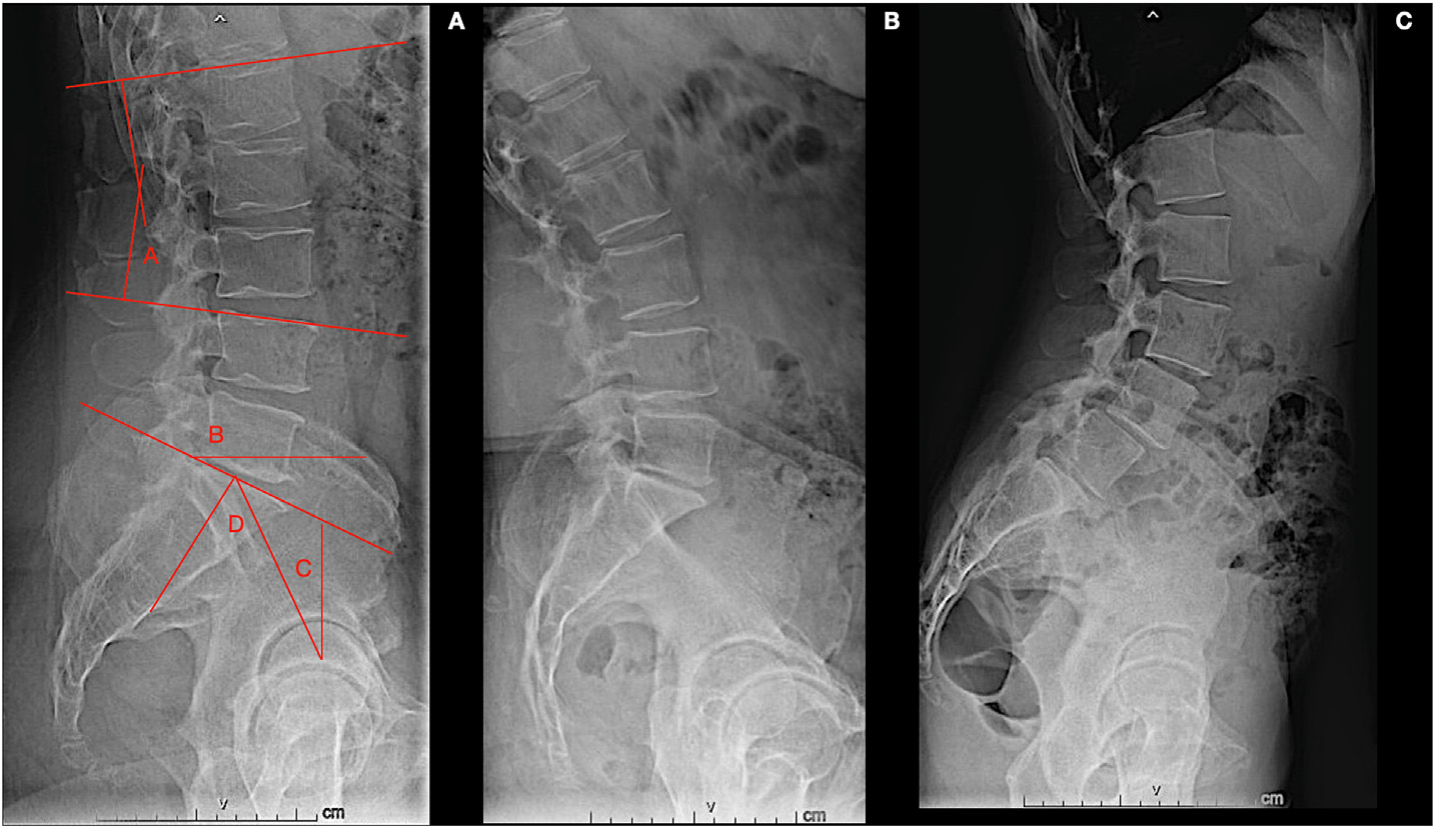
The figure shows the main Sagittal balance parameters used for the study (part A) namely Lumbar lordosis (red A), Sacral slope (red B), Pelvic Tilt (red C), Pelvic incidence (red D) and as can be seen in the X-ray images in maximum extension (part B) and maximum flexion (part C) of the patients, they can vary even significantly.

**Fig. 3:**
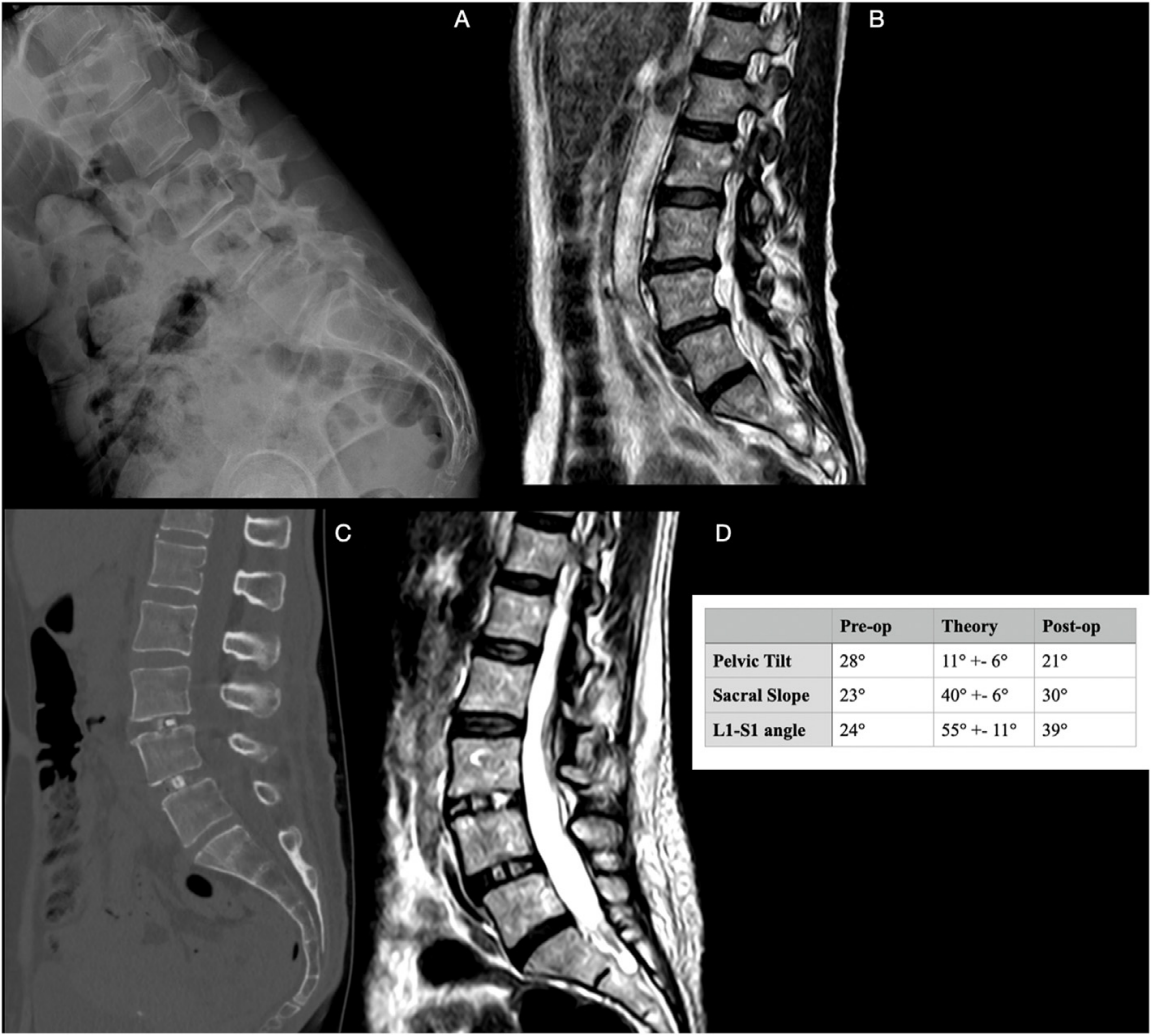
We report the clinical case of a 57-year-old woman with chronic low back pain and degenerative discopathy with L4-L5 listhesis (standing Rx A, and preoperative MRI, B) who benefited from a significant improvement in sagittal balance parameters after stabilization surgery with double MS-TLIF L3-L4-l5 with lordotic cages (CT scan after 1 month, C and MRI control after 6 months).

The analysis of these literature data shows sparse and variable evidence. It highlights how clarification and greater understanding of this argument are still needed, evoking the need for methodologically correct and high-level studies.^89^ Most of the works in the literature are retrospective, generally monocentric, based on small populations, and often address the topic evaluation partially. Indeed, the generation of standards based on scientific evidence remains very difficult for degenerative lumbar spine pathology. However, evaluating data collected on a sufficiently large population in a prospective, uniform, and methodologically correct manner could allow highlighting and underlining some associations. This future multicentric study is based on the possibility of comparing a heterogeneous population by pathology and different surgical technical options on some homogeneous clinical and anatomic-radiological measures (Fig. 2). The data analysis will contribute to understanding the value that global lumbar and segmental lordosis, distribution of lordosis, pelvic tilt, and PI-LL mismatch may have, as independent factors, on clinical outcome in lumbar degenerative pathology and on the occurrence of adjacent segment disease. Consequently, it will focus on and enhance at least some rational aspects of lumbar arthrodesis, such as, in particular, the need to adopt surgical strategies aimed at restoring segmental lordosis and correcting the sagittal profile (Fig. 3). Carrying on the follow-up for several years, the study will finally provide information on long-term evolution, particularly on the occurrence of symptomatic adjacent level degeneration.

## Disclosure

This study is not sponsored, and there are no conflicts of interest within the steering committee or for single investigators in front of the final results. The study is dedicated to the memory of Vincenzo Di Stefano, an excellent friend, person, and vertebral neurosurgeon.
